# Glasgow Royal Infirmary

**Published:** 1832-10

**Authors:** 


### GLASGOW ROYAL INFIRMARY.

### *Cases of Abscess of the Prostate Gland.**

CASE I. *Abscess of the Prostate, which was discharged by the Urethra; Cure.*

W. H., ætat. thirty-six, admitted December 9th, 1831. Complained of frequent, difficult, and painful micturition, with pain in loins, and dull uneasiness, with occasional throbbing in perinæo. These symptoms commenced two months before, and continued for three weeks, until the abscess burst, and the matter, unmixed with the urine, was discharged by the urethra. He slept ill; the tongue was loaded, the thirst urgent, and the pulse accelerated. On introducing a catheter, it produced severe pain at the neck of bladder, where a good deal of irregularity was felt; and, on examination per anum, the prostate gland was found enormously enlarged, but smooth and soft.

The treatment, which consisted of the frequent application of leeches to the perinæum, small doses of Oleum Ricini every second

* Macfarlane's Clinical Reports of the Glasgow Royal Infirmary.

morning, the hip-bath, and an occasional suppository at night, was persisted in for about three weeks, during which time the symptoms gradually decreased, the purulent discharge was arrested, and the difficult micturition disappeared.

This was a distinct case of phlegmonous inflammation of the prostate, which ended in suppuration, and could not be traced to any obvious cause. When there is a free communication between the abscess and the urethra, a simple straining effort will, in many cases, be sufficient to expel along the penis a considerable quantity of pus, unmixed with urine; but in general, the discharge in quantities takes place only when the patient empties his bladder; and then it may either precede or follow this evacuation, or the matter may be so mixed with the urine as to render it turbid immediately on its being discharged.

When the enlargement of the prostate is great, and especially when the abscess involves the middle lobe, there is always more or less difficulty in expelling the urine. This sometimes amounts to complete retention, and requires the introduction of a catheter; but this ought to be employed only in the most urgent circumstances, as the frequent introduction, or continued retention, of the instrument, will be found highly injurious.

When suppuration is fairly established, which may either be in the substance of the gland itself, or in the cellular texture which surrounds it, and unites its lobes together, it is but seldom we can succeed in detecting its existence, by manual examination. I have only met with one case in which, on introducing the finger into the rectum, distinct fluctuation was felt in the tumor: it was large, and projected considerably into the cavity of the bowel. This was punctured three times with a trocar, and a cure accomplished. The pain during the expulsion of the feces was most acute, and there was distressing tenesmus, from a sensation as if there was a hard body lodged in the rectum; but the pains in, or the impediment to, the evacuation of the bladder were less urgent than usual.

The following case was under treatment at the same time, and in the same ward with the last patient, but the disease was more severe and protracted.

CASE II. *Abscess of the Prostate; Retention of Urine; Cure.*

W. P., æt. twenty, admitted December 7th, 1831. Was seized six weeks previously, after a horse had fallen on him, with acute pain abut the neck of the bladder, stretching to the glans, and accompanied by frequent and painful micturition, tenesmus, and violent throbbing in the perinæum. During the last three weeks, he has continued to discharge large quantities of pus, separate from and mixed with the urine. The epididymis of the left testicle was hard, swollen, and painful when handled, without the gland itself being much affected. The prostate gland was much enlarged, and intolerant of pressure, both when this was applied through the rectum, or externally to the lower part of the perinæum.

The antiphlogistic treatment was adopted: leeches were repeatedly applied to the perinæum, followed by hot fomentations, the hip-bath, warm emollient and anodyne enemata, gentle laxatives, and a suspensory bandage to the testicle. By a continuance of this practice, and by keeping him closely in a recumbent position, the symptoms gradually diminished, and on the 20th he was dismissed, by his own desire, nearly well. He was again admitted on the 25th, when it appeared that the day before, after acute throbbing pain in the perinæum, and almost complete obstruction of urine, a copious discharge of pus took place suddenly from the urethra, and produced partial relief; but he still complained of painful and difficult micturition, every attempt to empty his bladder giving rise to tenesmus, and a desire to go to stool. The prostate was felt to be larger than formerly, especially its right lobe.—Hirudin. xviij. perin. Ol. Ricini. Vesper. cluniluv.

26th. Pain and difficulty in passing urine still continue, but tenesmus has abated; discharge of pus moderate.—Rept. Hirud. et iterum vesp. descend. in baln. tepid.

January 12th. Urine has been free of pus for the last three days; but he has been feverish, and complaining of rigors, pain in the perinæum, tenesmus, and an inability to expel his urine, which has been retained for ten hours. The bladder was felt greatly distended above the pubes, and the swelling of the prostate was ascertained, on examination per anum, to have considerably augmented. After using a hip-bath and an anodyne enema, I found it necessary, on account of the urgency of the symptoms, to draw off the urine with a catheter. When the instrument was passed as far as the prostate, it produced excruciating pain: the muscles of the perinæum were spasmodically excited, and the progress of the instrument arrested. After retaining it in this position for about five minutes, and attempting to pass it into the bladder, I had only advanced its point a few lines, when there was discharged by it fully two ounces of pus. It then slipped readily into the bladder, and gave exit to nearly two pounds of urine. Its introduction was not again necessary.

16th. Calls to void urine vary in frequency from one quarter of an hour to two hours. Complains only when expelling the last drops, and there is less pus discharged.—Sum. Tinct. Mur. Ferri gtt. xx. ter indies.

30th. The pain and difficulty in micturition have increased, and there is more pus in the urine, which frequently escapes involuntarily. Prostate continues enlarged, and there is throbbing in perinæo.—Omit. Tk. Ferri; Sum. Aq. Potassæ gtt. xxx. ter quotidie; Hirudin. xviij. perinæo; Baln. tepid. Enema emollien.

The symptoms gradually subsided, and he was dismissed, cured, on the 12th of February.

When irritation at the neck of the bladder is great, the testicle becomes often affected. In the last case, the inflammation seemed to have extended along the cord, producing hardness and thickening of the epididymis, without the gland becoming involved. This irritability of the prostatic portion of the urethra sometimes continues long after the abscess has closed, and is the cause of painful and impeded micturition. For this, in addition to the usual local and soothing treatment, I have experienced great advantage from small doses of the liquor potassæ.

When phlegmonous inflammation of the prostate gland occurs in old and enfeebled subjects, and especially when it is the consequence of stricture of the urethra, the destruction which it occasions is sometimes so extensive as to lead to a fatal termination, by giving rise to urinary extravasation. In such cases the prostatic disease is almost always the consequence of acute inflammation of the mucous membrane of the neck of the bladder, and origin of the urethra; which inflammation may exist for a considerable time before the gland or its cellular connexions become implicated.

